# Assessment of Human BSE Risks Through the Use of Cattle-Derived MBM^*1^ in Chicken, Pig, and Others Feed (Prions)

**DOI:** 10.14252/foodsafetyfscj.D-25-00029

**Published:** 2025-12-19

**Authors:** Food Safety Commission of Japan

## Abstract

Food Safety Commission of Japan (FSCJ) conducted a risk assessment regarding the use of so-called “*cattle-derived MBM*” as raw material in feed intended for ***chickens, pigs, and others*** in response to a request of the Ministry of Agriculture, Forestry and Fisheries (MAFF). As far as ***the current risk mitigation measures against BSE*** are implemented, BSE prions are highly unlikely to be accumulated in the cattle, sheep, and goat parts which would be used as raw materials of feed for ***chickens, pigs, and others***. There are negligible occurrence of *cattle-derived MBM* to be fed to cattle and other ruminants, as long as the Japanese current risk mitigation measures against feeding *cattle-derived MBM* to ruminants continue to be abided. Furthermore, oral transmission of BSE prions to ***chickens, pigs, and others*** is unlikely to occur, based on accumulated scientific findings. The risk of human infection with BSE is considered to be highly unlikely. FSCJ thus concluded negligible adverse human health-effects of foods from chickens, pigs, and others, in Japan’s circumstances where *cattle-derived MBM* is used as raw materials for feed these specified animals.

## Conclusion in Brief

Food Safety Commission of Japan (FSCJ) conducted a risk assessment regarding the use of so-called “*cattle-derived MBM”* as raw material in feed intended for chickens, pigs, and others^*2^ in response to a request of the Ministry of Agriculture, Forestry and Fisheries (MAFF). Published scientific literatures and MAFF submitted documents were used on the current assessment.

The assessment was conducted based on investigations and deliberations. The committee reviewed and discussed previous FSCJ assessments, and also Japanese risk mitigation measures and their outcomes. Thus, the safety of *cattle-derived MBM*, risks of human infection after consuming ***chickens, pigs, and others*** fed *cattle-derived MBM*, and also the possibility of contaminations during the production and erroneous feedings were evaluated.

Specifically, the risk of human infection with BSE prion and variant Creutzfeldt-Jakob disease (vCJD) is considered from human consumption of the followings:

1. ***Chickens, pigs, and others***, fed feed containing *cattle-derived MBM*

2. Cattle, sheep, or goats fed feed containing *cattle-derived MBM*, (which might occur as a result of cross-contamination between feed intended for cattle^*3^and feed intended for ***chickens, pigs, and others***).

The raw materials (*cattle-derived MBM*), expected to be used in feed for ***chickens, pigs, and others***, do not contain fallen stock of cattle, sheep or goats, or the specified risk materials (SRM). There are no observations affecting FSCJ’s previous evaluations of meat and offal of cattle, nor of meat and offal of sheep and goats so far investigated. Based on these observations, BSE prions are highly unlikely to be accumulated in the cattle, sheep, and goat parts which would be used as raw materials of feed for ***chickens, pigs, and others***, as far as the current risk mitigation measures against BSE^*4^ are implemented.

To evaluate the risk of human infection with BSE from food products of *cattle-derived MBM* consuming ***chickens, pigs, and others***, susceptibility and transmissibility of prions into these animals were assessed. There is no scientific data on the infection of pigs and poultry and transmission of BSE prions in their natural states. An infection experiment demonstrated that pigs were not susceptible to oral exposure to BSE prions, although susceptible to the parenteral exposures. It is recognized that natural infection does not occur through oral exposure. There is no report of prion diseases in horse up to now. The dissimilar structure of horse prion protein may link to the resistance to transmissible prion diseases. No new finding is available to affect these established views.

Transmission of BSE prions to ***chickens, pigs, and others*** is unlikely to occur, based on scientific findings on the susceptibility and transmissibility of prions in the aforementioned target animals potentially fed *cattle-derived MBM*. Transmission of BSE to humans through these target animals fed *cattle-derived MBM*, is also highly unlikely in considering the safety of the raw materials (*cattle-derived MBM*).

The results of regulatory authority’s inspections on the current preventative measures against cross-contamination risks of feed, and other risk mitigation measures underwent deliberation. FSCJ judged that the current risk mitigation measures are effective.

There are negligible occurrence of *cattle-derived MBM* to be fed to cattle and other ruminants, as long as the Japanese current risk mitigation measures against feeding *cattle-derived MBM* to ruminants continue to be abided. Combined with the above-mentioned safety of raw materials (*cattle-derived MBM*), the risk of human infection with BSE is considered to be highly unlikely.

FSCJ concluded negligible adverse human health-effects of foods from ***chickens, pigs, and others***, in the circumstances using *cattle-derived MBM,* as raw materials for feed intended for **these specified animals.**

## Note

^*1^ Term “***cattle-derived MBM***” means processed animal proteins which contain meat-and-bone meal (MBM), hydrolyzed protein, steamed bone meal, blood meal and plasma protein derived from cattle, sheep and goats (ruminants). “Protein meal” is used, instead of MBM currently.

^*2^ Term “***chickens, pigs, and others***” corresponds chickens, quails, pigs and horses (non-ruminants).

^*3^ Term “***feed intended for cattle***” includes feed intended for cattle, sheep, goats and deer (ruminants).

^*4^ “***The current risk mitigation measures against BSE”*** are the following actions stipulated and enforced by the Government of Japan under the relevant laws and regulations for controlling BSE:

1. Disposal of bovine SRM* at slaughterhouses and meat processing plants (elimination from the food chain and feed chain).

* SRM includes: tonsils and distal ileum from cattle of all ages, head (excluding tongue, cheek meat, skin and tonsils), spinal cord and vertebral column from cattle over 30 months of age (as of May 2024).

2. Ban on feeding animal-derived MBM to ruminants.
